# Patterns of intron sequence evolution in *Drosophila *are dependent upon length and GC content

**DOI:** 10.1186/gb-2005-6-8-r67

**Published:** 2005-07-27

**Authors:** Penelope R Haddrill, Brian Charlesworth, Daniel L Halligan, Peter Andolfatto

**Affiliations:** 1 Institute of Evolutionary Biology, School of Biological Sciences, University of Edinburgh, Edinburgh EH9 3JT, UK; 2 Section of Ecology, Behavior and Evolution, Division of Biological Sciences, University of California San Diego, La Jolla, CA 92093, USA

## Abstract

**Background:**

Introns comprise a large fraction of eukaryotic genomes, yet little is known about their functional significance. Regulatory elements have been mapped to some introns, though these are believed to account for only a small fraction of genome wide intronic DNA. No consistent patterns have emerged from studies that have investigated general levels of evolutionary constraint in introns.

**Results:**

We examine the relationship between intron length and levels of evolutionary constraint by analyzing inter-specific divergence at 225 intron fragments in *Drosophila melanogaster *and *Drosophila simulans*, sampled from a broad distribution of intron lengths. We document a strongly negative correlation between intron length and divergence. Interestingly, we also find that divergence in introns is negatively correlated with GC content. This relationship does not account for the correlation between intron length and divergence, however, and may simply reflect local variation in mutational rates or biases.

**Conclusion:**

Short introns make up only a small fraction of total intronic DNA in the genome. Our finding that long introns evolve more slowly than average implies that, while the majority of introns in the *Drosophila *genome may experience little or no selective constraint, most intronic DNA in the genome is likely to be evolving under considerable constraint. Our results suggest that functional elements may be ubiquitous within longer introns and that these introns may have a more general role in regulating gene expression than previously appreciated. Our finding that GC content and divergence are negatively correlated in introns has important implications for the interpretation of the correlation between divergence and levels of codon bias observed in *Drosophila*.

## Abstract 2

An analysis of inter-specific divergence in 225 intron fragments in *Drosophila melanogaster *and *D. simulans *reveals a strongly negative correlation between intron length and divergence and intron divergence and GC content. This suggests that most intronic DNA is evolving under considerable constraint.

## Background

Non-coding DNA makes up a large proportion of the genomes of most eukaryotes, yet little is known about its functional significance and the forces affecting its evolution. The identification of functional regions of the genome has tended to concentrate on coding DNA, yet the recent shift in focus towards non-coding DNA has revealed that introns and intergenic sequences may be subject to considerable levels of selective constraint, implying that they contain functional elements [1-6]. No consistent patterns have emerged from the relatively few studies that have thus far investigated levels of constraint on intron DNA sequences; some studies conclude that such DNA is evolving under little or no selective constraint, while others find considerable levels of constraint (for examples, see [3,7-10]). Moreover, the mode of evolution for such types of sequence is still unclear.

Several recent studies have attempted to estimate the proportion of sites within introns that is subject to selective constraint. For example, Jareborg *et al. *[11] estimate that 23% of intronic sites in mouse-rat genome comparisons are evolutionarily conserved. Similarly, Shabalina and Kondrashov [12] estimate (conservatively) that 17% of nucleotide sites within introns are selectively constrained between *Caenorhabditis elegans *and *Caenorhabditis briggsae*; this was at least in part due to their function in splicing, because constraint appeared to be higher at the edges of introns. Likewise, Bergman and Kreitman [3] estimate that 22-26% of non-coding sequences (intergenic and intronic) are highly constrained between *Drosophila melanogaster *and *Drosophila virilis*. In contrast to these studies, Halligan *et al. *[9] found that most intronic sites (excluding those necessary for correct splicing) in *Drosophila *were evolving approximately 17% faster than fourfold synonymous sites. They concluded that these sites were effectively evolving free from selective constraint. The discrepancies among previous studies suggest that no clear conclusions can yet be drawn regarding the levels of selective constraint in non-coding intronic DNA.

Intron size is one possible factor that may explain these conflicting results. Comeron and Kreitman [13] and others have noted an asymmetrical distribution of intron lengths in *D. melanogaster*; a large number of short introns clustered around a minimal intron length and a broader distribution of longer introns (median intron size of 86 base-pairs (bp), mean intron size of 1411 bp; [14]). Based on multi-species data for 15 introns (13 short and 2 long), Parsch [15] showed that there were significantly fewer substitutions per site in the two longer introns. He suggested that this pattern may be due to the presence of a greater number of regulatory elements that are subject to purifying selection in longer introns.

If regulatory elements occur frequently in introns, and these are of some minimal size, it follows that size may be an important factor in intron evolution. In agreement with this prediction, Marais *et al. *[16] noted a marginally significant (*P *= 0.03) negative correlation between intron divergence and size for first introns (but not other introns) in the dataset of Halligan *et al. *[9]. Marais *et al. *[16] suggested that this correlation between divergence and length may be expected for first introns because they are on average two times longer than other introns [17] and also tend to contain more known regulatory elements, at least in mammals [8]. Because the dataset used consisted mostly of short introns, it is unclear whether the pattern they observed is specific to first introns (due to an association between first introns and regulatory elements) and whether the relationship between divergence and size is primarily driven by the fact that first introns are longer. Here we revisit the relationship between intron length and evolutionary constraint (as measured by levels of divergence between *D. melanogaster *and *D. simulans*) by combining published data for 225 intron fragments sampled from a much broader distribution of intron lengths and positions within genes.

## Results and discussion

### Levels of divergence are correlated with intron length

**Figure 1:**
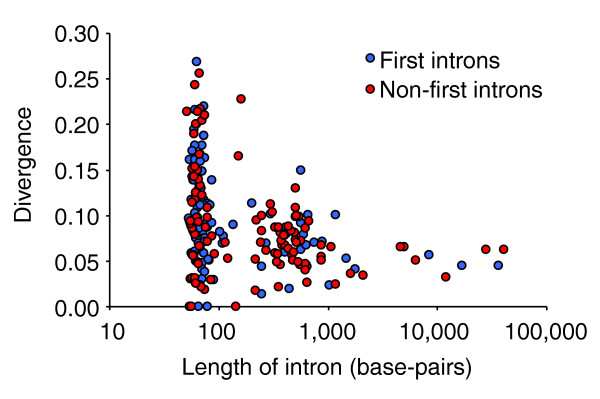
The relationship between intron length and the level of divergence between *D. melanogaster *and *D. simulans *for the combined dataset of 225 introns. A significantly negative correlation is found for all introns (Spearman correlation coefficient *R*_*s *_= -0.388, *P *< 10^-4^), first introns (*R*_*s *_= -0.451, *P *< 10^-4^) and non-first introns (*R*_*s *_= -0.304, *P *< 10^-4^).

We investigated levels of divergence at a total of 225 introns (a mixture of complete short introns and several hundred base-pair fragments of longer introns) scattered across the *Drosophila *genome. The relationship between intron length and nucleotide divergence for all complete introns and intron fragments surveyed is shown in Figure 1. A strongly negative correlation between intron length and divergence is apparent (Spearman correlation coefficient *R*_*s *_= -0.388, *P *< 10^-4^). We also divided the data into two size classes based on the median intron size of 86 bp in *Drosophila *[14]; small (≤86 bp) introns and large (>86 bp) introns. The large intron class showed significantly lower divergences than the small intron class (Wilcoxon two-sample test statistic W = 17079.5, *P *< 10^-4^). The correlation between intron length and divergence is somewhat weaker, but still significant within the longer intron class (*R*_*s *_= -0.278, *P *= 0.006).

It has been noted that introns harbouring regulatory elements tend to be first introns [6,8], and that first introns tend to be longer in *Drosophila *[17]. Thus a relationship between intron size and divergence might only be expected for first introns [16]. Indeed, previous studies have failed to find evidence of constraint outside first introns [16,18]. In Figure 1, we show that the strong correlation between divergence and intron length is not specific to first introns (first introns *R*_*s *_= -0.451, *P *< 10^-4^; non-first introns *R*_*s *_= -0.304, *P *< 10^-4^). Mean divergences were not significantly different between first and non-first introns when compared within short and long size classes (Table 1). These results suggest that regulatory elements may be common enough across all longer introns that constraint is independent of the position of an intron within a gene.

**Figure 2:**
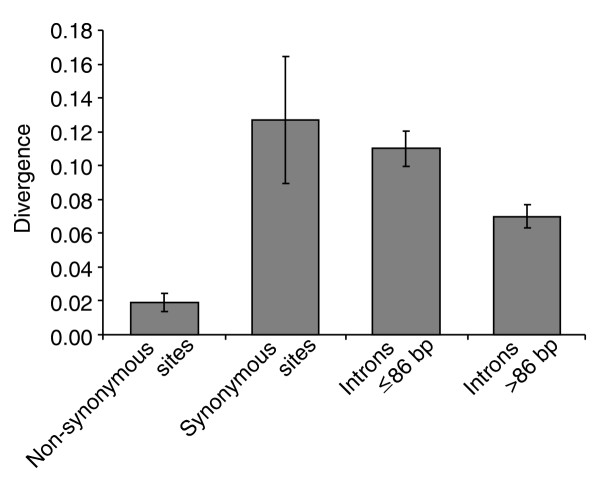
Mean divergences for non-synonymous sites, synonymous sites and both small and large introns. Mean levels of divergence between *D. melanogaster *and *D. simulans *for non-synonymous and synonymous sites of coding data, introns ≤86 bp and introns >86 bp. Error bars indicate two standard errors. Synonymous site divergence is significantly greater than large (Wilcoxon two-sample test statistic W = 7745.5, *P *< 10^-4^) but not small (W = 15115.5, *P *= 0.6173) intron divergences. Small intron divergence is significantly greater than large intron divergence (W = 17079.5, *P *< 10^-4^).

While this is strong evidence for evolutionary constraint on longer introns, short introns do not appear to evolve much more slowly than synonymous sites in *Drosophila*. To illustrate this, Figure 2 shows average divergence estimates (with two standard errors) for synonymous sites from 102 coding regions [19] compared to those for the small (≤86 bp) and large (>86 bp) size classes of introns. Average divergence at non-synonymous sites [19] is also shown for comparison. Synonymous site divergence is significantly higher than levels of divergence for large introns (Wilcoxon two-sample W = 7745.5, *P *< 10^-4^) but not small introns (Wilcoxon two-sample W = 15115.5, *P *= 0.617). This finding is consistent with the conclusions of Halligan *et al. *[9] that introns and synonymous sites evolve at similar rates, given that their dataset contained few long introns. One half of the introns in the genome are less than 86 base-pairs long, but these comprise only about 5% of total intronic DNA in the genome [14]. Thus, ironically, while the majority of introns in the *Drosophila *genome may be evolving under little or no selective constraint, most intronic DNA in the genome is likely to be evolving under considerable constraint.

### Divergence and base composition of introns

**Figure 3:**
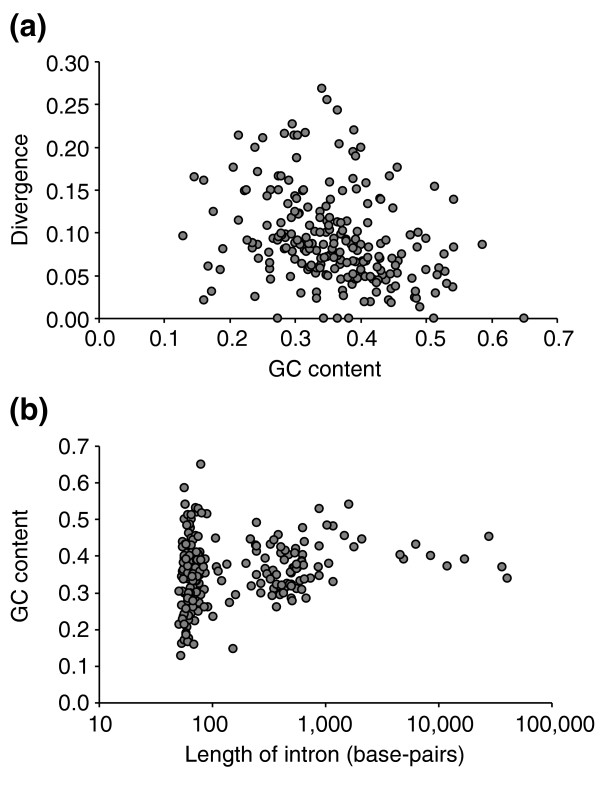
The relationship between intron fragment GC content and both divergence and length. **(a) **The relationship between GC content of intron fragments and divergence between *D. melanogaster *and *D. simulans *(Spearman correlation coefficient *R*_*s *_= -0.345, *P *< 10^-4^). **(b) **The relationship between GC content of intron fragments and intron length (*R*_*s *_= 0.237, *P *< 10^-3^).

Introns are more AT-rich than synonymous sites in *Drosophila *[20] (Table 1). Could lower levels of divergence then be an artefact of local GC content? There is a significantly negative relationship between divergence and GC content in the intron dataset (*R*_*s *_= -0.345, *P *< 10^-4^) (Figure 3a), and a significantly positive relationship between intron length and GC content (*R*_*s *_= 0.237, *P *< 10^-3^) (Figure 3b). The partial correlation coefficient for divergence versus length, controlling for GC content, is -0.132 (95% bootstrap confidence interval: -0.192/-0.089). The partial correlations for divergence versus GC content (controlling for length) and GC content versus length (controlling for divergence) were -0.292 (-0.410/-0.168) and 0.030 (-0.037/0.120), respectively. These results suggest that the relationship between intron length and divergence is not a confounding effect of GC content, despite the negative correlation between divergence and GC content.

Similar to the pattern we observe in introns, a negative association between synonymous site substitution rates and GC content at the third position of codons has previously been noted in *Drosophila *[21] and in mammals [22]. This pattern at synonymous sites has been cited as evidence of selection for codon usage bias, as preferred codons are usually GC rich [21,23]; however, selection on codon usage obviously cannot explain the same pattern in introns. The negative relationship between divergence and GC content in introns might instead reflect local variation in the extent of mutational rates or biases [22,24], or the effects of biased gene conversion favouring GC over AT, which mimics the effect of selection in favour of GC nucleotides [25].

The possible role of mutational bias can be examined using the following method. It follows from the standard model of drift and reversible mutation that, if AT mutates to GC at rate *u *and GC mutates to AT at rate *ku *the equilibrium frequency of GC for neutral sites (neglecting polymorphic sites) is approximated by *p *= 1/(1 + *k*), and the equilibrium rate of substitutions is *K *= 2*uk*/(1+*k*) [26,27]. This yields the relation *K *= 2*u*(1 - *p*), so that the equilibrium rate of substitution is negatively and linearly related to GC content. This formula predicts that the intercept (divergence at zero GC content) is equal to the absolute value of the slope, and so this hypothesis is testable. The regression coefficient of divergence on GC content in the complete dataset is -0.180 (-0.254/-0.106), and the corresponding intercept is 0.157 (0.115/0.163), which at first sight is consistent with the hypothesis that variation in the level of the mutational bias parameter, *k*, is sufficient to account for the relation between divergence and GC content.

The relationship between divergence and length, however, makes the above test problematic, in view of the wide variation in intron length. If only the 127 short introns (length ≤ 86 bp) are used, which are much more uniform in length, the regression of divergence on GC content is almost unchanged at -0.116 (-0.207/-0.023), and the intercept is 0.150 (0.142/0.162). Note, however, that there is a significant partial correlation of 0.166 (0.041/0.345) between GC content and length for short introns, but not for long introns, so there is still a residual relation between length and GC content in short introns. While we cannot rule out the possibility that biased gene conversion and/or selection in favour of GC versus AT explains the relationship between GC content and divergence, our analysis suggests that variation in mutational bias may be sufficient. If this process also explains the relationship between synonymous site divergence and GC content, tests for selection on codon bias based on negative correlations between codon bias and divergence (recently discussed by Bierne and Eyre-Walker [28] and Dunn *et al*. [29]) lose their force. These have been criticized on other theoretical grounds by Eyre-Walker and Bulmer [26].

### The density of functional elements in introns

The correlation analyses strongly suggest that longer introns show lower levels of divergence, and that this is not simply caused by mutational rate differences related to GC content, although other sources of mutation rate differences cannot of course be ruled out. So why might longer introns be subject to higher levels of constraint? Introns are known to contain regulatory elements (for examples, see [30,31], and see [32] for a recent review of the mammalian literature), so it is possible that longer introns are more constrained because they contain more of these elements.

Are putative regulatory elements in longer introns discrete entities (such as clusters of binding sites for transcription factors), or is this regulatory function more diffuse? If intronic regulatory elements occur in clusters, surrounded by unconstrained regions, we might expect to find higher levels of divergence in the short, several hundred base-pair regions of very long introns (such as those surveyed here), compared to intermediate-sized introns, provided that they have similar total amounts of regulatory sequences. The rationale for this is that, if constrained regulatory elements are clustered into one region, short fragments of very long introns would be unlikely to coincide by chance with a functional element, whereas similarly sized regions from introns of intermediate length would be more likely to coincide with such elements. Such clustering is possible, given that transcription factor binding sites and regulatory elements can range in size from a few base-pairs up to several hundred base-pairs (for examples, see [33-36]). If the proportion of regulatory sequence is similar in long and intermediate introns, however, no difference in mean divergence is expected, but clustering would cause a higher variance in divergence in very long versus intermediate-length introns (after removing the binomial sampling variance). If regulatory elements in introns are widely dispersed, however, there is no reason to expect greater means or variances of divergence in fragments from very long introns. In fact, the mean divergence for the small number of intron fragments from introns longer than 4,500 bp is 0.054 (SE = 0.004, n = 9). This is significantly smaller than for the small (≤86 bp) intron class (mean divergence = 0.110, n = 127, Wilcoxon two-sample W = 252, *P *= 0.001) and marginally significantly lower than for introns of intermediate size (between 87 bp and 4,500 bp: mean divergence = 0.072, n = 89, W = 4494, *P *= 0.044). The non-binomial standard deviation in divergence is estimated to be 0.0056 for the very long introns, compared with 0.023 for the 38 intermediate-sized ones for which fragments at least 20 bp shorter than the introns were used for estimating divergence (this ensures that both classes represent samples rather than complete sequences). This is the opposite pattern to what is expected with strong clustering of regulatory sequences. Levels of constraint, and thus the density of putatively funtional regulatory elements, therefore appear to be relatively uniform across longer introns.

A uniform density of regulatory functions is unexpected if these often involve clusters of, for example, transcription factor binding sites. However, it might be expected, for example, if the regulatory functions of introns often involve the formation of complex secondary structures. Evidence suggesting that intron sequence and length affects the secondary structure of precursor messenger RNA (pre-mRNA) is accumulating. If this secondary structure plays a regulatory role, it is likely to be conserved. Several studies have found evidence for epistatic selection on introns to maintain pre-mRNA secondary structure [37-39], and there is also evidence for a functional role of RNA secondary structure in splicing [40,41] and gene expression [42,43]. For example, Chen and Stephan [44] found that mutations disrupting a hairpin structure in intron 1 of the *D. melanogaster Adh *gene reduce splicing efficiency and decrease production of the *Adh *protein. These authors show that compensatory mutations that restore the secondary structure result in a mutant indistinguishable from the wild type in splicing efficiency and protein production. A hairpin structure in the second intron of this gene also shows striking structural conservation across ten species in three sub-genera of *Drosophila *[45]. Our finding that the density of constrained sequences does not appear to be a function of intron length (within the long intron class) suggests that pre-mRNA secondary structure may be a more common mechanism mediating gene regulation than discrete regulatory elements such as intronic transcriptional enhancers.

## Conclusion

Most introns in *Drosophila *are relatively short, but these short introns make up only a small fraction of total intronic DNA in the genome. We demonstrate that levels of selective constraint are higher with increasing intron length. Thus, while the majority of introns in the *Drosophila *genome may be evolving under little or no selective constraint, the majority of intronic DNA in the genome is likely to be evolving under considerable constraint. We also find that the density of functionally important elements within longer introns does not appear to depend on their length. This suggests that functional elements may be ubiquitous within longer introns and that these introns may have a more general role in regulating gene expression than previously appreciated, possibly via the formation of pre-mRNA secondary structures. This pattern contrasts with that found in mammals, where constraint does not appear to be a function of intron length [46] (A Kondrashov, personal communication). An unexpected corollary of our study is the finding of a negative correlation between divergence and GC content in introns. This finding implies that a similar pattern observed for synonymous sites in *Drosophila *may reflect mutational biases rather than selection for codon usage.

## Materials and methods

### Introns

We combined data from three recent studies of complete introns or several hundred base-pair fragments of longer introns located on the X chromosome of *D. melanogaster*. Halligan *et al. *[9] compiled previously published data for *D. melanogaster *and *D. simulans *sequences for each of 163 introns. We combined these data with introns surveyed in *D. melanogaster *and *D. simulans *by Glinka *et al. *[47]. All the Glinka *et al. *[47] intron fragments were compared to the DNA sequence of the *D. melanogaster *genome [48]. Ten of these intron fragments were removed from the analysis because they contained exonic or 5'/3' untranslated region sequences. The alignments for a further 12 of the Glinka *et al. *[47] fragments were trimmed to remove small quantities of exonic or untranslated region sequences. The final Glinka *et al. *[47] dataset used in the analysis therefore contained 53 intron fragments (details on request to PR Haddrill). To this we added nine more intron fragments surveyed by Haddrill *et al. *[49]. For consistency with Halligan *et al. *[9], we realigned these sequences with the program MCALIGN, using the insertion-deletion frequency model defined for *Drosophila *intronic DNA [50,51]. Divergence estimates per site and the GC content of introns were generated for each alignment (excluding the 6 bp/16 bp at the 5'/3' end of the intron, which include bases that are constrained because they are necessary for correct splicing) using the DnaSP software package (Version 4) [52], which corrects divergence values for multiple hits using the Jukes-Cantor equation [53]. The use of divergence as a proxy for constraint is appropriate, because the level of selective constraint in a sequence will directly affect the divergence between two species; highly constrained sequences will show little divergence, whereas sequences under little or no selective constraint will accumulate differences more rapidly. Sites overlapping alignment gaps were excluded from the count of total base-pairs. The total length of each intron was determined using the DNA sequence of the *D. melanogaster *genome [48]. The mean total intron length across the entire dataset was 936.5 bp and the mean length of the fragments of introns analyzed here was 230.2 bp.

Because we did not analyse the entire length of all of the introns included in this study, we were unable to investigate whether intron lengths vary substantially between *D. melanogaster *and *D. simulans*. Previous evidence suggests that intron lengths are unlikely to differ to any great extent between the two species, however, and that transitions between the short and long intron size class are rare [15,20,54].

Partial moment correlation coefficients and least-squares regression coefficients were calculated by the standard formulae, and their significance assessed by bootstrapping over loci 1,000 times to obtain their resampling distributions [55].

### Coding regions

As a comparison for levels of divergence at intron sites, we used synonymous site divergences from 102 genes compiled by Betancourt and Presgraves [19]. Single-pass sequenced ESTs from this same study were not included in the analysis. Estimates of synonymous site divergences calculated using the Nei and Gojobori [56] correction were kindly provided by A Betancourt. Divergence estimates for synonymous sites based on *D. melanogaster *- *D. simulans *alignments for 35 additional X-linked coding regions were identical, and did not differ significantly from divergence estimates for fourfold degenerate sites (P Andolfatto, unpublished data). Several previous studies have documented a positive relationship between exon length and synonymous site divergences [57-59]. This relationship is in the opposite direction to that which would be expected if there were some (unknown) factor co-varying with gene length and neutral divergence that was responsible for the negative association between intron length and intron divergence. Non-synonymous site divergences from the same 102 genes compiled by Betancourt and Presgraves [19] (kindly provided by A Betancourt) were also used in Figure 2 for visual comparison with synonymous and intron sites; as expected, these are smaller than the other values, consistent with strong selection against most amino acid substitutions.

### Effects of sex linkage

As our data come from three different sources, we investigated possible biases relating to how and why the data were collected. In particular, the studies of Haddrill *et al*. [49] and Glinka *et al. *[47] surveyed intron fragments from longer introns on the X chromosome, whereas the data of Halligan *et al. *[9] contains mostly short introns from all chromosomes. We note a significant difference between autosomal versus X-linked introns in both levels of divergence (Wilcoxon two-sample W = 13502.5, *P *= 0.006) and GC content (W = 13211.5, *P *= 0.005). When comparing within size classes (≤86 bp versus >86 bp), however, levels of divergence are not significantly different between autosomal and X-linked introns, and GC content is significantly different for the short intron class, but not the long intron class. The negative correlation between intron length and divergence holds for autosomal and X-linked introns separately (autosomes, Spearman *R*_*s *_= -0.261, *P *= 0.006; X-linked, Spearman *R*_*s *_= -0.403, *P *< 10^-4^) as does the negative relationship between GC content and divergence (autosomes, Spearman *R*_*s *_= -0.281, *P *= 0.003; X-linked, Spearman *R*_*s *_= -0.371, *P *< 10^-4^). The differences in levels of divergence and GC content between autosomal and X-linked introns, therefore, cannot explain the observed relationships between intron length versus divergence and GC content versus divergence.

## Additional data files

The following additional data are available with the online version of this paper. Additional data file 1 is an Excel file listing all introns analyzed. Additional data files 2, 3 and 4 conatain alignments of the Glinka *et al. *[47], Haddrill *et al. *[49] and Halligan *et al. *[9] data, respectively. Additional data file 5 contains programs written to carry out partial moment correlations, least-squares regressions and bootstrapping procedures and the data used for these analyses.

## Supplementary Material

Additional File 1 An Excel file listing all introns analyzed Click here for file

Additional File 2 Alignments of the Glinka *et al. *[47] data Click here for file

Additional File 3 Alignments of the Haddrill *et al. *[49] data Click here for file

Additional File 4 Alignments of the Halligan *et al. *[9] data Click here for file

Additional File 5 Programs written to carry out partial moment correlations, least-squares regressions and bootstrapping procedures and the data used for these analyses Click here for file

## Figures and Tables

**Table 1:** Mean divergence and GC content values for each class of DNA

	Divergence			GC Content		
	All	Short*	Long*	All	Short*	Long*
Introns						
All	0.093 (0.004)	0.110 (0.005)	0.070 (0.003)	0.357 (0.006)	0.345 (0.009)	0.371 (0.007)
First	0.101 (0.005)	0.114^† ^(0.006)	0.072^† ^(0.006)	0.361 (0.010)	0.352^† ^(0.013)	0.383^† ^(0.011)
Non-first	0.085 (0.005)	0.105^† ^(0.009)	0.069^† ^(0.004)	0.352 (0.007)	0.337^† ^(0.012)	0.365^† ^(0.008)
Synonymous sites	0.127 (0.019)			0.654 (0.014)		

Values are mean (standard error). *Introns were divided into two classes based on the median intron length (86 bp) [14]: short, ≤86 bp; long, >86 bp. ^†^Divergence and GC content values did not differ between first and non-first introns when compared within long and short size classes.
