# Supplementary material for: Patterns of intron sequence evolution in Drosophila are dependent upon length and GC content
Source: Genome Biol. 2005 Jul 27;6(8):R67. doi: 10.1186/gb-2005-6-8-r67 (PMC1273634; doi:10.1186/gb-2005-6-8-r67)
Supplement: Additional File 5 — Programs written to carry out partial moment correlations, least-squares regressions and bootstrapping procedures and the data used for these analyses [file gb-2005-6-8-r67-S5.zip › regression_data.doc]

225

51	0.21429	0.214

51	0.21429	0.304

53	0.09677	0.129

54	0.09375	0.266

54	0	0.344

54	0.16129	0.161

55	0.09091	0.439

55	0.09375	0.359

55	0.0303	0.424

55	0.09091	0.303

55	0.09091	0.227

56	0.08824	0.235

56	0.11765	0.338

56	0.08824	0.235

56	0	0.364

56	0.05882	0.338

56	0	0.382

57	0.08571	0.586

57	0.05714	0.5

57	0.15152	0.394

57	0.14286	0.3

57	0.08571	0.243

57	0.08571	0.4

57	0.03125	0.172

57	0.17143	0.243

57	0.11429	0.214

58	0.08571	0.386

58	0.13889	0.542

58	0.05556	0.431

58	0.08333	0.542

59	0.19444	0.389

59	0.08108	0.189

59	0.05714	0.186

59	0.13889	0.431

59	0.14286	0.257

59	0.18919	0.392

60	0.24324	0.365

60	0.15789	0.408

60	0.17647	0.206

60	0.07895	0.395

60	0.03125	0.484

61	0.15385	0.513

61	0.16667	0.278

61	0.16129	0.29

61	0.11765	0.353

61	0.06061	0.167

61	0.10256	0.372

61	0.21622	0.284

62	0.1	0.488

62	0.15	0.275

62	0.125	0.338

62	0.05	0.35

62	0.15	0.225

62	0.075	0.463

62	0.15	0.263

62	0.025	0.238

62	0.125	0.175

62	0.2	0.238

62	0.125	0.3

63	0.09756	0.28

63	0.08108	0.284

63	0.08333	0.486

63	0.26829	0.341

63	0.2	0.4

64	0.10811	0.257

64	0.09756	0.476

64	0.04878	0.415

64	0.09524	0.274

64	0.15	0.313

64	0.21429	0.298

65	0.04651	0.395

65	0.12821	0.449

65	0.06977	0.244

65	0.09302	0.5

65	0.13953	0.302

65	0	0.512

65	0.13953	0.43

66	0.16667	0.274

66	0.25581	0.349

66	0.08571	0.343

67	0.06667	0.389

67	0.13333	0.411

68	0.12195	0.305

68	0.13333	0.289

68	0.21739	0.315

68	0.16667	0.444

68	0.15909	0.352

68	0.02381	0.333

68	0.06522	0.261

68	0.11111	0.278

69	0.13043	0.348

69	0.02128	0.16

70	0.14894	0.223

71	0.14894	0.372

71	0.04762	0.381

71	0.20408	0.367

71	0.17647	0.456

71	0.12245	0.306

71	0.04082	0.531

71	0.0303	0.333

72	0.08163	0.286

73	0.05882	0.52

73	0.11765	0.294

73	0.1875	0.302

73	0.11111	0.411

73	0.05882	0.333

73	0.1875	0.302

73	0.22	0.39

74	0.16327	0.388

74	0.07143	0.345

75	0.01887	0.406

75	0.21053	0.25

75	0.07547	0.528

75	0.03774	0.453

76	0.08696	0.326

77	0.09091	0.273

78	0.07143	0.411

78	0.11321	0.349

78	0.08696	0.359

79	0.10811	0.351

79	0.09804	0.294

79	0	0.649

80	0.05172	0.388

81	0.05085	0.517

84	0.11111	0.37

85	0.02941	0.309

86	0.07692	0.26

87	0.13846	0.354

87	0.09231	0.392

90	0.02941	0.515

91	0.05797	0.261

102	0.08163	0.235

108	0.06977	0.448

110	0.07692	0.369

114	0.07059	0.359

121	0.05263	0.332

135	0.09009	0.378

143	0	0.273

152	0.16505	0.146

160	0.22727	0.295

201	0.11364	0.379

217	0.01775	0.447

218	0.05155	0.446

220	0.09444	0.317

241	0.07009	0.43

245	0.01345	0.491

247	0.1	0.348

248	0.08293	0.412

248	0.04425	0.427

269	0.06911	0.299

270	0.06173	0.325

297	0.11211	0.365

297	0.10156	0.395

307	0.10385	0.352

326	0.0873	0.431

329	0.05882	0.337

339	0.04878	0.293

345	0.05814	0.36

350	0.02167	0.443

354	0.08667	0.312

368	0.04605	0.262

369	0.08127	0.322

374	0.07192	0.349

384	0.06704	0.457

394	0.07843	0.317

399	0.07849	0.295

404	0.0593	0.425

405	0.06739	0.408

412	0.08684	0.321

418	0.08036	0.314

431	0.08815	0.3

437	0.08187	0.389

438	0.06361	0.31

439	0.01966	0.415

462	0.08312	0.326

468	0.05631	0.319

477	0.05057	0.403

487	0.05932	0.322

493	0.08095	0.357

500	0.12953	0.319

503	0.07359	0.354

504	0.09829	0.285

505	0.10879	0.322

512	0.09942	0.278

516	0.07002	0.367

537	0.04843	0.407

542	0.09836	0.37

551	0.04563	0.419

560	0.07249	0.365

560	0.09234	0.378

562	0.14928	0.312

568	0.0625	0.368

599	0.07952	0.333

618	0.0472	0.384

620	0.08624	0.308

621	0.03993	0.387

622	0.04618	0.385

633	0.06714	0.403

635	0.04639	0.478

640	0.02623	0.374

648	0.04437	0.439

653	0.10067	0.338

673	0.09417	0.285

744	0.07018	0.339

872	0.06763	0.37

876	0.05438	0.529

876	0.05085	0.347

890	0.0716	0.428

1033	0.02335	0.485

1074	0.0651	0.379

1169	0.02466	0.482

1171	0.10092	0.33

1472	0.05254	0.456

1602	0.03636	0.541

1780	0.04114	0.424

2112	0.03409	0.447

4620	0.06567	0.404

4947	0.06571	0.392

6330	0.05046	0.433

8477	0.05627	0.4

12043	0.03204	0.373

16890	0.0452	0.392

27707	0.06207	0.453

36241	0.04525	0.37

40532	0.06263	0.339
