# Supplementary material for: Patterns of intron sequence evolution in Drosophila are dependent upon length and GC content
Source: Genome Biol. 2005 Jul 27;6(8):R67. doi: 10.1186/gb-2005-6-8-r67 (PMC1273634; doi:10.1186/gb-2005-6-8-r67)
Supplement: Additional File 5 — Programs written to carry out partial moment correlations, least-squares regressions and bootstrapping procedures and the data used for these analyses [file gb-2005-6-8-r67-S5.zip › Regression.doc]

C	REGRESSION PROGRAM WITH BOOTSTRAPPING

c	partial correlations and multiple regressions are also calculated

DIMENSION L(1000),X(1000),Y(1000),idbstr(1000),A(1000),B(1000),R(1000),ls(1000),bylb(1000)

DIMENSION lb(1000),xb(1000),yb(1000),yminb(1000),ymaxb(1000),ry(1000),xs(1000),byxb(1000)

DIMENSION ryc(1000),yminbc(1000),ymaxbc(1000),rpb1(1000),rpb2(1000),rpb3(1000),yintb(1000)

PARAMETER (numg=32)

C	.. Scalars in Common ..

INTEGER a1,a1vw,a1w,a2,a2vw,a2w,m1,m2,iseed1,iseed2

C	.. Arrays in Common ..

INTEGER cg1(numg),cg2(numg),ig1(numg),ig2(numg),lg1(numg),

+	lg2(numg)

C	.. Common blocks ..

COMMON /ranfs/m1,m2,a1,a2,a1w,a2w,a1vw,a2vw,ig1,ig2,lg1,lg2,cg1,

+	cg2,qanti

CHARACTER*9 FINP

CHARACTER*9 FOUT

WRITE (9,*) 'Input file?'

READ (9,*) FINP

OPEN (2,FILE=FINP)

WRITE (9,*) 'Output file?'

READ (9,*) FOUT

OPEN (1,FILE=FOUT)

REWIND 1

WRITE (1,*) 'Regression of divergence on GC content'

WRITE (1,*) 'Partial correlations and multiple regression using intron length also found '

WRITE (1,*) ''

READ (2,*) nb1

WRITE (1,*) 'Number of observations',nb1

WRITE (1,*) ''

do 5 i=1,nb1

READ (2,*) L(i),Y(i),X(i)

xs(i)=X(i)

ls(i)=L(i)

5	write (1,*) L(i),X(i),Y(i)

CLOSE(2)

WRITE (1,*) ''

c	L is length, X is GC content, Y is divergence

AMGM=0

SGEN=0

SSGEN=0

SSMGEN=0

SSGENM=0

sl=0

sxl=0

syl=0

ssl=0

do 10 i=1,nb1

sl=sl+L(i)

ssl=ssl+L(i)**2

sxl=sxl+L(i)*X(i)

syl=syl+L(i)*Y(i)

AMGM=AMGM+Y(i)

SSGENM=SSGENM+Y(i)**2

SGEN=SGEN+X(i)

SSGEN=SSGEN+X(i)**2

10	SSMGEN=SSMGEN+X(i)*Y(i)

c	calculates statistics for unweighted regression

x	pause

iregdf=nb1-2

const=nb1

SSGEN=SSGEN-SGEN**2/const

SSGENM=SSGENM-(AMGM**2)/const

SSMGEN=SSMGEN-AMGM*SGEN/const

BMGEN=SSMGEN/SSGEN

AMGEN=(AMGM-BMGEN*SGEN)/const

CORR=SSMGEN/SQRT(SSGEN*SSGENM)

VARB=(SSGENM*(1.0-CORR**2))/(SSGEN*(iregdf))

VARA=(SSGENM*(1.0-CORR**2)/iregdf)*((1.0/const)+((SGEN/const)**2)/SSGEN)

SEB=SQRT(VARB)

SEA=SQRT(VARA)

xm=SGEN/const

ym=AMGM/const

alm= sl/const

ssl=ssl-sl**2/const

sxl=sxl-sl*SGEN/const

syl=syl-sl*AMGM/const

rxl=sxl/SQRT(ssl*SSGEN)

ryl=syl/SQRT(ssl*SSGENM)

rp1=(CORR-rxl*ryl)/((1-rxl**2)*(1-ryl**2))

rp2=(ryl-rxl*CORR)/((1-rxl**2)*(1-CORR**2))

rp3=(rxl-ryl*CORR)/((1-ryl**2)*(1-CORR**2))

c	correlations of x and y on l and partial correlations

vxm=SSGEN/(const*(nb1-1))

vym=SSGENM/(const*(nb1-1))

sexm=SQRT(vxm)

seym=SQRT(vym)

WRITE (1,*) ''

WRITE (1,*) 'Unweighted regression coefficient',BMGEN

WRITE (1,*) 'D.f. ',iregdf,' s.e. ',SEB

WRITE (1,*) ''

WRITE (1,*) 'Intercept ',AMGEN

WRITE (1,*) 's.e.',SEA

WRITE (1,*) ''

WRITE (1,*) 'Mean GC ',xm,' s.e ',sexm

WRITE (1,*) ''

WRITE (1,*) 'Mean intron length ',ym,' s.e ',seym

WRITE (1,*) ''

write (1,*) 'Correlation div/GC',CORR

write (1,*) ''

write (1,*) 'Correlation div/length',ryl

write (1,*) ''

write (1,*) 'Correlation GC/length',rxl

write (1,*) ''

write (1,*) 'Partial correlation coefficient div/GC',rp1

write (1,*) ''

write (1,*) 'Partial correlation coefficient div/length',rp2

write (1,*) ''

write (1,*) 'Partial correlation coefficient GC/length',rp3

write (1,*) ''

call Sort1(xs,ls,nb1)

xmin=xs(1)

xmax=xs(nb1)

write (1,*) 'xmin,xmax',xmin,xmax

write (1,*) 'lmin,lmax ',ls(1),ls(nb1)

c	maximum and minimum X values

ymin=AMGEN+BMGEN*xmin

ymax=AMGEN+BMGEN*xmax

c	predicted maximum and minimum Y values

write (1,*) ''

write (1,*) 'predicted max. and min. Y values from ordinary regression',ymin,ymax

write (1,*) ''

byl=SQRT((SSGENM/ssl))*(ryl-CORR*rxl)/(1-rxl**2)

byx=SQRT((SSGENM/SGEN))*(CORR-ryl*rxl)/(1-rxl**2)

c	multiple regression coefficients

yint=ym-byx*xm

c	predicted y value at mean length and zero GC

write (1,*) 'Multiple regression coefficients: byl ',byl,' byx ',byx

write (1,*) 'Intercept for mr of y on x at mean l ',yint

yminc=ym+byl*(ls(1)-alm)+byx*(xmin-xm)

ymaxc=ym+byl*(ls(nb1)-alm)+byx*(xmax-xm)

c	multiple regression predictions

write (1,*) 'predicted max. and min. Y values from multiple regression',yminc,ymaxc

write (1,*) ''

c	Start of bootstrapping section

200	write (9,*) 'no. of bootstraps?'

read (9,*) nbstr

if(nbstr.lt.2) go to 500

c	no bootstrapping asked for

write (1,*) ''

write (1,*) 'no. of bootstraps ',nbstr

write (9,*) 'seeds?'

read(9,*) iseed1,iseed2

call setall(iseed1,iseed2)

write (1,*) ''

write (1,*) ''

300	do 310 nrep=1,nbstr

call Boot(nb1,idbstr,iseed1,iseed2)

c	resets statistics for bootstrapped regression analysis

AMGM=0

SGEN=0

SSGEN=0

SSMGEN=0

SSGENM=0

sl=0

sxl=0

syl=0

ssl=0

do 100 j=1,nb1

i1=idbstr(j)

x	write (9,*) 'i1',i1

lb(j)=L(i1)

xb(j)=X(i1)

yb(j)=Y(i1)

xs(j)=xb(j)

ls(j)=lb(j)

x	write (9,*) xb(j),yb(j)

c	assigns bootstrapped variables

sl=sl+lb(j)

ssl=ssl+lb(j)**2

sxl=sxl+lb(j)*xb(j)

syl=syl+lb(j)*yb(j)

AMGM=AMGM+yb(j)

SSGENM=SSGENM+yb(j)**2

SGEN=SGEN+xb(j)

SSGEN=SSGEN+xb(j)**2

100	SSMGEN=SSMGEN+xb(j)*yb(j)

c	calculates bootstrapped statistics for unweighted regression

SSGEN=SSGEN-SGEN**2/const

SSGENM=SSGENM-(AMGM**2)/const

SSMGEN=SSMGEN-AMGM*SGEN/const

B(nrep)=SSMGEN/SSGEN

A(nrep)=(AMGM-BMGEN*SGEN)/const

R(nrep)=SSMGEN/SQRT(SSGEN*SSGENM)

xm=SGEN/const

ym=AMGM/const

alm=sl/const

ssl=ssl-sl**2/const

sxl=sxl-sl*SGEN/const

syl=syl-sl*AMGM/const

rxl=sxl/SQRT(ssl*SSGEN)

ryl=syl/SQRT(ssl*SSGENM)

rpb1(nrep)=(R(nrep)-rxl*ryl)/((1-rxl**2)*(1-ryl**2))

rpb2(nrep)=(ryl-rxl*R(nrep))/((1-rxl**2)*(1-R(nrep)**2))

rpb3(nrep)=(rxl-ryl*R(nrep))/((1-ryl**2)*(1-R(nrep)**2))

c	correlations of x and y on l and partial correlations

bylb(nrep)=SQRT((SSGENM/ssl))*(ryl-CORR*rxl)/(1-rxl**2)

byxb(nrep)=SQRT((SSGENM/SGEN))*(CORR-ryl*rxl)/(1-rxl**2)

c	multiple regression coefficients

yintb(nrep)=ym-byxb(nrep)*xm

c	intercept for y at mean l

call Sort1(xs,ls,nb1)

xmin=xs(1)

xmax=xs(nb1)

xmin=xs(1)

xmax=xs(nb1)

c	maximum and minimum X values

yminb(nrep)=A(nrep)+B(nrep)*xmin

ymaxb(nrep)=A(nrep)+B(nrep)*xmax

ry(nrep)=yminb(nrep)/ymaxb(nrep)

c	predicted maximum and minimum bootstrapped Y values and their ratio

yminbc(nrep)=ym+bylb(nrep)*(ls(1)-alm)+byxb(nrep)*(xmin-xm)

ymaxbc(nrep)=ym+bylb(nrep)*(ls(nb1)-alm)+byxb(nrep)*(xmax-xm)

ryc(nrep)=yminbc(nrep)/ymaxbc(nrep)

c	multiple regression predictions

310	continue

write(1,*) 'Distributional statistics '

write (1,*) ''

write (1,*) 'Regression coefficient of Y on X '

call Sort3(B,nbstr)

write (1,*) ''

write (1,*) 'Intercept of Y on X '

call Sort3(A,nbstr)

write (1,*) ''

write (1,*) 'Correlation of Y on X '

call Sort3(R,nbstr)

write (1,*) ''

write (1,*) 'Partial correlation of Y and X '

call Sort3(rpb1,nbstr)

write (1,*) ''

write (1,*) 'Partial correlation of Y and L '

call Sort3(rpb2,nbstr)

write (1,*) ''

write (1,*) 'Partial correlation of X and L '

call Sort3(rpb3,nbstr)

write (1,*) ''

write (1,*) 'Multiple regression of Y and L '

call Sort3(bylb,nbstr)

write (1,*) ''

write (1,*) 'Multiple regression of Y and X '

call Sort3(byxb,nbstr)

write (1,*) ''

write (1,*) 'Multiple regression intercept of Y on X at mean L'

call Sort3(yintb,nbstr)

write (1,*) 'Predicted min. value of Y '

call Sort3(yminb,nbstr)

write (1,*) ''

write (1,*) 'Predicted max. value of Y '

call Sort3(ymaxb,nbstr)

write (1,*) ''

write (1,*) 'Ratio min/max for pred. Y '

call Sort3(ry,nbstr)

write (1,*) ''

write (1,*) 'Predicted mult. reg. min. value of Y '

call Sort3(yminbc,nbstr)

write (1,*) ''

write (1,*) 'Predicted mult. reg. max. value of Y '

call Sort3(ymaxbc,nbstr)

write (1,*) ''

write (1,*) 'Ratio mult. reg. min/max for pred. Y '

call Sort3(ryc,nbstr)

write (1,*) ''

CLOSE(1)

500	END

Subroutine Boot(nl,idbstr,iseed1,iseed2)

c	bootstraps over genes

DIMENSION idbstr(1000)

do 50 j=1,nl

rn=ranf()

c	Random number between 0 and 1

xgn=rn*nl

do 60 i=1,nl

if (xgn.le.i) then

go to 80

end if

60	continue

c	i is original locus corresponding to bootstrap locus j

80	idbstr(j)=i

c	idbstr(j) is identity of bootstrap genotype j

x	write(9,*) 'bs genotype',j,'true genotype ',i

50	continue

x	pause

RETURN

END

Subroutine Sort3(x,nb)

Dimension x(1000)

nr1=nb-1

do 40 i=1,nr1

i2=i+1

do 50 j1=i2,nb

x1=x(j1)

if(x(j1).lt.x(i)) then

x(j1)=x(i)

x(i)=x1

end if

50	continue

40	continue

c	sorts bootstrapped values into ordered list

an05=nb*0.025

if(an05.lt.1) then

n05=1

go to 100

end if

n05=nint(an05)

100	xg05=x(n05)

write(1,*) '5% value ',xg05

an01=nb*0.005

if(an01.lt.1) then

n01=1

go to 110

end if

n01=nint(an01)

110	x01=x(n01)

write (1,*) '1% value ',x01

nb2=nb-1

an95=nb*0.975

if(an95.gt.nb2) then

n95=nb

go to 120

end if

n95=nint(an95)

120	x95=x(n95)

write (1,*) '95% value ',x95

an99=nb*0.995

if(an99.gt.nb2) then

n99=nb

go to 130

end if

n99=nint(an99)

130	x99=x(n99)

write (1,*) '99% value',x99

write(1,*) ''

RETURN

END

Subroutine Sort1(x,l,nb)

Dimension x(1000),l(1000)

nr1=nb-1

do 40 i=1,nr1

i2=i+1

do 50 j1=i2,nb

x1=x(j1)

l1=l(j1)

if(x(j1).lt.x(i)) then

x(j1)=x(i)

l(j1)=l(i)

x(i)=x1

l(i)=l1

end if

50	continue

40	continue

c	sorts into ordered list

RETURN

END

REAL FUNCTION ranf()

C**********************************************************************

C

C	REAL FUNCTION RANF()

C	RANDom number generator as a Function

C

C	Returns a random floating point number from a uniform distribution

C	over 0 - 1 (endpoints of this interval are not returned) using the

C	current generator

C

C**********************************************************************

C	.. External Functions ..

INTEGER ignlgi

EXTERNAL ignlgi

ranf = ignlgi()*4.656613057E-10

RETURN

END

INTEGER FUNCTION ignlgi()

C**********************************************************************

C

C	INTEGER FUNCTION IGNLGI()

C	GeNerate LarGe Integer

C

C	Returns a random integer following a uniform distribution over

C	(1, 2147483562) using the current generator.

C

C**********************************************************************

C	.. Parameters ..

INTEGER numg

PARAMETER (numg=32)

C	..

INTEGER a1,a1vw,a1w,a2,a2vw,a2w,m1,m2

INTEGER cg1(numg),cg2(numg),ig1(numg),ig2(numg),lg1(numg),

+	lg2(numg)

LOGICAL qanti(numg)

C	..

C	.. Local Scalars ..

INTEGER curntg,k,s1,s2,z

LOGICAL qqssd

C	..

C	.. External Functions ..

LOGICAL qrgnin

EXTERNAL qrgnin

C	..

C	.. External Subroutines ..

EXTERNAL getcgn,inrgcm,rgnqsd,setall

C	..

COMMON /ranfs/m1,m2,a1,a2,a1w,a2w,a1vw,a2vw,ig1,ig2,lg1,lg2,cg1,

+	cg2,qanti

C	.. Save statement ..

SAVE /ranfs/

C	..

IF (.NOT. (qrgnin())) CALL inrgcm()

CALL rgnqsd(qqssd)

IF (.NOT. (qqssd)) CALL setall(1234567890,123456789)

C

C	Get Current Generator

C

CALL getcgn(curntg)

s1 = cg1(curntg)

s2 = cg2(curntg)

k = s1/53668

s1 = a1* (s1-k*53668) - k*12211

IF (s1.LT.0) s1 = s1 + m1

k = s2/52774

s2 = a2* (s2-k*52774) - k*3791

IF (s2.LT.0) s2 = s2 + m2

cg1(curntg) = s1

cg2(curntg) = s2

z = s1 - s2

IF (z.LT.1) z = z + m1 - 1

IF (qanti(curntg)) z = m1 - z

ignlgi = z

RETURN

END

SUBROUTINE setall(iseed1,iseed2)

C**********************************************************************

C

C	SUBROUTINE SETALL(ISEED1,ISEED2)

C	SET ALL random number generators

C

C	Sets the initial seed of generator 1 to ISEED1 and ISEED2. The

C	initial seeds of the other generators are set accordingly, and

C	all generators states are set to these seeds.

C

C**********************************************************************

C	.. Parameters ..

INTEGER numg

PARAMETER (numg=32)

C	..

C	.. Scalar Arguments ..

INTEGER iseed1,iseed2

LOGICAL qssd

INTEGER a1,a1vw,a1w,a2,a2vw,a2w,m1,m2

INTEGER cg1(numg),cg2(numg),ig1(numg),ig2(numg),lg1(numg),

+	lg2(numg)

LOGICAL qanti(numg)

C	..

C	.. Local Scalars ..

INTEGER g,ocgn

LOGICAL qqssd

C	..

C	.. External Functions ..

INTEGER mltmod

LOGICAL qrgnin

EXTERNAL mltmod,qrgnin

C	..

C	.. External Subroutines ..

EXTERNAL getcgn,initgn,inrgcm,setcgn

C	..

C	.. Common blocks ..

COMMON /ranfs/m1,m2,a1,a2,a1w,a2w,a1vw,a2vw,ig1,ig2,lg1,lg2,cg1,

+	cg2,qanti

C	..

C	.. Save statement ..

SAVE /ranfs/,qqssd

C	..

C	.. Data statements ..

DATA qqssd/.FALSE./

C	..

qqssd = .TRUE.

CALL getcgn(ocgn)

IF (.NOT. (qrgnin())) CALL inrgcm()

ig1(1) = iseed1

ig2(1) = iseed2

CALL initgn(-1)

DO 10,g = 2,numg

ig1(g) = mltmod(a1vw,ig1(g-1),m1)

ig2(g) = mltmod(a2vw,ig2(g-1),m2)

CALL setcgn(g)

CALL initgn(-1)

10 CONTINUE

CALL setcgn(ocgn)

RETURN

ENTRY rgnqsd(qssd)

C**********************************************************************

C

C	SUBROUTINE RGNQSD

C	Random Number Generator Query SeeD set?

C

C	Returns (LOGICAL) QSSD as .TRUE. if SETALL has been invoked,

C	otherwise returns .FALSE.

C

C**********************************************************************

qssd = qqssd

RETURN

END

SUBROUTINE getsd(iseed1,iseed2)

C**********************************************************************

C

C	SUBROUTINE GETSD(G,ISEED1,ISEED2)

C	GET SeeD

C

C	Returns the value of two integer seeds of the current generator

C

C**********************************************************************

C	.. Parameters ..

INTEGER numg

PARAMETER (numg=32)

C	..

C	.. Scalar Arguments ..

INTEGER iseed1,iseed2

INTEGER a1,a1vw,a1w,a2,a2vw,a2w,m1,m2

INTEGER cg1(numg),cg2(numg),ig1(numg),ig2(numg),lg1(numg),

+	lg2(numg)

LOGICAL qanti(numg)

C	..

C	.. Local Scalars ..

INTEGER g

C	..

C	.. External Functions ..

LOGICAL qrgnin

EXTERNAL qrgnin

EXTERNAL getcgn

COMMON /ranfs/m1,m2,a1,a2,a1w,a2w,a1vw,a2vw,ig1,ig2,lg1,lg2,cg1,

+	cg2,qanti

C	..

C	.. Save statement ..

SAVE /ranfs/

C	Abort unless random number generator initialized

IF (qrgnin()) GO TO 10

WRITE (*,*) ' GETSD called before random number generator ',

+ ' initialized -- abort!'

STOP ' GETSD called before random number generator initialized'

10	CALL getcgn(g)

iseed1 = cg1(g)

iseed2 = cg2(g)

RETURN

END

SUBROUTINE getcgn(g)

INTEGER g

C**********************************************************************

C

C	SUBROUTINE GETCGN(G)

C	Get GeNerator

C

C	Returns in G the number of the current random number generator

C**********************************************************************

C

INTEGER curntg,numg

SAVE curntg

PARAMETER (numg=32)

DATA curntg/1/

C

g = curntg

RETURN

ENTRY setcgn(g)

C**********************************************************************

C

C	SUBROUTINE SETCGN( G )

C	Set GeNerator

C

C	Sets the current generator to G. All references to a generat

C	are to the current generator.

C**********************************************************************

C

C	Abort if generator number out of range

C

IF (.NOT. (g.LT.0.OR.g.GT.numg)) GO TO 10

WRITE (*,*) ' Generator number out of range in SETCGN:',

+ ' Legal range is 1 to ',numg,' -- ABORT!'

STOP ' Generator number out of range in SETCGN'

10	curntg = g

RETURN

END

LOGICAL FUNCTION qrgnin()

C**********************************************************************

C

C	LOGICAL FUNCTION QRGNIN()

C	Q Random GeNerators INitialized?

C

C	A trivial routine to determine whether or not the random

C	number generator has been initialized. Returns .TRUE. if

C	it has, else .FALSE.

C

C**********************************************************************

C	.. Scalar Arguments ..

LOGICAL qvalue

C	..

C	.. Local Scalars ..

LOGICAL qinit

C	..

C	.. Entry Points ..

LOGICAL qrgnsn

C	..

C	.. Save statement ..

SAVE qinit

C	..

C	.. Data statements ..

DATA qinit/.FALSE./

C	..

C	.. Executable Statements ..

qrgnin = qinit

RETURN

ENTRY qrgnsn(qvalue)

C**********************************************************************

C

C	LOGICAL FUNCTION QRGNSN( QVALUE )

C	Q Random GeNerators Set whether iNitialized

C

C	Sets state of whether random number generator is initialized

C	to QVALUE.

C

C	This routine is actually an entry in QRGNIN, hence it is a

C	logical function. It returns the (meaningless) value .TRUE.

C

C**********************************************************************

qinit = qvalue

qrgnsn = .TRUE.

RETURN

END

SUBROUTINE inrgcm()

C**********************************************************************

C

C	SUBROUTINE INRGCM()

C	INitialize Random number Generator CoMmon

C

C

C	Function

C

C

C	Initializes common area for random number generator. This saves

C	the nuisance of a BLOCK DATA routine and the difficulty of

C	assuring that the routine is loaded with the other routines.

C

C**********************************************************************

C	.. Parameters ..

INTEGER numg

PARAMETER (numg=32)

INTEGER a1,a1vw,a1w,a2,a2vw,a2w,m1,m2

INTEGER cg1(numg),cg2(numg),ig1(numg),ig2(numg),lg1(numg),

+	lg2(numg)

LOGICAL qanti(numg)

C	..

C	.. Local Scalars ..

INTEGER i

LOGICAL qdum

LOGICAL qrgnsn

EXTERNAL qrgnsn

COMMON /ranfs/m1,m2,a1,a2,a1w,a2w,a1vw,a2vw,ig1,ig2,lg1,lg2,cg1,

+	cg2,qanti

C	..

C	.. Save statement ..

SAVE /ranfs/

C

m1 = 2147483563

m2 = 2147483399

a1 = 40014

a2 = 40692

a1w = 1033780774

a2w = 1494757890

a1vw = 2082007225

a2vw = 784306273

DO 10,i = 1,numg

qanti(i) = .FALSE.

10	CONTINUE

C

C	Tell the world that common has been initialized

qdum = qrgnsn(.TRUE.)

RETURN

END

SUBROUTINE initgn(isdtyp)

C**********************************************************************

C

C	SUBROUTINE INITGN(ISDTYP)

C	INIT-ialize current G-e-N-erator

C

C	Reinitializes the state of the current generator

C

C**********************************************************************

C	.. Parameters ..

INTEGER numg

PARAMETER (numg=32)

C	..

C	.. Scalar Arguments ..

INTEGER isdtyp

INTEGER a1,a1vw,a1w,a2,a2vw,a2w,m1,m2

INTEGER cg1(numg),cg2(numg),ig1(numg),ig2(numg),lg1(numg),

+	lg2(numg)

LOGICAL qanti(numg)

C	..

C	.. Local Scalars ..

INTEGER g

LOGICAL qrgnin

INTEGER mltmod

EXTERNAL qrgnin,mltmod

EXTERNAL getcgn

COMMON /ranfs/m1,m2,a1,a2,a1w,a2w,a1vw,a2vw,ig1,ig2,lg1,lg2,cg1,

+	cg2,qanti

C	..

C	.. Save statement ..

SAVE /ranfs/

C	..

C	Abort unless random number generator initialized

IF (qrgnin()) GO TO 10

WRITE (*,*) ' INITGN called before random number generator ',

+ ' initialized -- abort!'

STOP ' INITGN called before random number generator initialized'

10 CALL getcgn(g)

IF ((-1).NE. (isdtyp)) GO TO 20

lg1(g) = ig1(g)

lg2(g) = ig2(g)

GO TO 50

20 IF ((0).NE. (isdtyp)) GO TO 30

CONTINUE

GO TO 50

C	do nothing

30 IF ((1).NE. (isdtyp)) GO TO 40

lg1(g) = mltmod(a1w,lg1(g),m1)

lg2(g) = mltmod(a2w,lg2(g),m2)

GO TO 50

40 STOP 'ISDTYP NOT IN RANGE'

50 cg1(g) = lg1(g)

cg2(g) = lg2(g)

RETURN

END

INTEGER FUNCTION mltmod(a,s,m)

C**********************************************************************

C

C	INTEGER FUNCTION MLTMOD(A,S,M)

C

C	Returns (A*S) MOD M

C

C**********************************************************************

C	.. Parameters ..

INTEGER h

PARAMETER (h=32768)

C	..

C	.. Scalar Arguments ..

INTEGER a,m,s

C	..

C	.. Local Scalars ..

INTEGER a0,a1,k,p,q,qh,rh

C	..

C	H = 2**((b-2)/2) where b = 32 because we are using a 32 bit

C	machine. On a different machine recompute H

C

IF (.NOT. (a.LE.0.OR.a.GE.m.OR.s.LE.0.OR.s.GE.m)) GO TO 10

WRITE (*,*) ' A, M, S out of order in MLTMOD - ABORT!'

WRITE (*,*) ' A = ',a,' S = ',s,' M = ',m

WRITE (*,*) ' MLTMOD requires: 0 < A < M; 0 < S < M'

STOP ' A, M, S out of order in MLTMOD - ABORT!'

10 IF (.NOT. (a.LT.h)) GO TO 20

a0 = a

p = 0

GO TO 120

20 a1 = a/h

a0 = a - h*a1

qh = m/h

rh = m - h*qh

IF (.NOT. (a1.GE.h)) GO TO 50

a1 = a1 - h

k = s/qh

p = h* (s-k*qh) - k*rh

30 IF (.NOT. (p.LT.0)) GO TO 40

p = p + m

GO TO 30

40 GO TO 60

50 p = 0

C

C	P = (A2*S*H)MOD M

C

60 IF (.NOT. (a1.NE.0)) GO TO 90

q = m/a1

k = s/q

p = p - k* (m-a1*q)

IF (p.GT.0) p = p - m

p = p + a1* (s-k*q)

70 IF (.NOT. (p.LT.0)) GO TO 80

p = p + m

GO TO 70

80 CONTINUE

90 k = p/qh

C

C	P = ((A2*H + A1)*S)MOD M

C

p = h* (p-k*qh) - k*rh

100 IF (.NOT. (p.LT.0)) GO TO 110

p = p + m

GO TO 100

110 CONTINUE

120 IF (.NOT. (a0.NE.0)) GO TO 150

C

C	P = ((A2*H + A1)*H*S)MOD M

C

q = m/a0

k = s/q

p = p - k* (m-a0*q)

IF (p.GT.0) p = p - m

p = p + a0* (s-k*q)

130 IF (.NOT. (p.LT.0)) GO TO 140

p = p + m

GO TO 130

140 CONTINUE

150 mltmod = p

C

RETURN

END
